# RapidPlan head and neck model: the objectives and possible clinical benefit

**DOI:** 10.1186/s13014-017-0808-x

**Published:** 2017-04-27

**Authors:** A. Fogliata, G. Reggiori, A. Stravato, F. Lobefalo, C. Franzese, D. Franceschini, S. Tomatis, P. Mancosu, M. Scorsetti, L. Cozzi

**Affiliations:** 1 Humanitas Research Hospital and Cancer Center, Radiotherapy and Radiosurgery Department, Milan, Rozzano Italy; 2 Department of Biomedical Sciences, Humanitas University, Milan, Rozzano Italy

**Keywords:** Knowledge based planning, RapidPlan, Head and Neck planning, VMAT, SIB

## Abstract

**Background:**

To evaluate a knowledge based planning model for RapidPlan (RP) generated for advanced head and neck cancer (HNC) patient treatments, as well its ability to possibly improve the clinical plan quality. The stability of the model was assessed also for a different beam geometry, different dose fractionation and different management of bilateral structures (parotids).

**Methods:**

Dosimetric and geometric data from plans of 83 patients presenting HNC were selected for the model training. All the plans used volumetric modulated arc therapy (VMAT, RapidArc) to treat two targets at dose levels of 69.96 and 54.45 Gy in 33 fractions with simultaneous integrated boost. Two models were generated, the first separating the ipsi- and contra-lateral parotids, while the second associating the two parotids to a single structure for training. The optimization objectives were adjusted to the final model to better translate the institutional planning and dosimetric strategies and trade-offs. The models were validated on 20 HNC patients, comparing the RP generated plans and the clinical plans. RP generated plans were also compared between the clinical beam arrangement and a simpler geometry, as well as for a different fractionation scheme.

**Results:**

RP improved significantly the clinical plan quality, with a reduction of 2 Gy, 5 Gy, and 10 Gy of the mean parotid, oral cavity and laryngeal doses, respectively. A simpler beam geometry was deteriorating the plan quality, but in a small amount, keeping a significant improvement relative to the clinical plan. The two models, with one or two parotid structures, showed very similar results. NTCP evaluations indicated the possibility of improving (NTCP decreasing of about 7%) the toxicity profile when using the RP solution.

**Conclusions:**

The HNC RP model showed improved plan quality and planning stability for beam geometry and fractionation. An adequate choice of the objectives in the model is necessary for the trade-offs strategies.

## Background

The radiotherapy treatment planning of head and neck cancer (HNC), especially for advanced stages, is a very complex and challenging process with severe trade-offs between target coverage and organs at risk (OAR) sparing. The advent of the new techniques, like intensity modulated radiotherapy (IMRT) and volumetric modulated arc therapy (VMAT), in parallel with the use of altered fractionation schemes like the simultaneous integrated boost (SIB), allowed to greatly improving the global conformal avoidance degree of the treatments. Nevertheless, the inherent complexity of the planning process, regardless from the technique applied, enhances the impact of two general problems: the skills of the individual planners might affect the quality of the treatment plans, and the institutional criteria, strategies or protocols applied to the plan acceptance phase are often insufficient to properly discriminate the dosimetric trade-offs per each individual patient. Therefore, inhomogeneous and possibly inconsistent criteria are applied within a single institute and more likely among different centres. Pragmatically, one of the main risks is to spend too long time and resources in the planning processes to obtain results that are not always consistent and of high quality.

The need of consistency in treatment planning drove many efforts aiming to develop processes and algorithms for automation and harmonization. Contouring/segmentation of OARs and target volumes and optimisation of dose distributions are the two main areas of investigation [1–6]. The concept of knowledge based planning (KBP) [7–9] is one example of the results of this hunt. In its conception, KBP is not simply a solution for automated treatment planning. With KBP, a number of optimal plans (judged to be the optimum) generated following the clinical criteria and appropriate trade-off requirements, are used to build and train a mathematical model capable to estimate OAR dose volume histograms (DVH) for any new patient. With this conceptual basis, any new patients planned via the application of the KBP method, should result with a plan whose quality falls within the boundaries of the set of plans used for the model training. RapidPlan (Varian Medical Systems, Palo Alto, CA) is the commercial KBP process implemented in the Varian Eclipse treatment planning system. Briefly, for a new patient, estimated DVHs for the OARs are generated by the application of the trained model and then translated into optimization objectives used by the inverse planning optimisation engines. The RapidPlan (RP) process was appraised in different anatomical sites: liver [10], head and neck [11], nasopharyngeal cancer patients [12], lung and prostate [13], breast [14], pelvis as prostate and cervical cancers [15], oesophagus [16], lung SBRT [17]. Those studies proved the possibility to build, train and apply a variety of models leading to improved quality of the treatment plans and better homogeneity of results. A critical point in the RP model configuration was shown to be related to the presence (or not) of outliers in the training set. Outliers are cases that could influence the generation of the model parameters and bias the results. In the absence of general rules, some criteria to better identify and manage these possible outliers were published by some groups [15, 18]. However, the RP model configuration process in all the needed steps is still to be fully understood.

The present work has manifold aims: *i*) to generate a model for advanced HNC planning and its subsequent validation, to prove if RP based plan can improve the clinical plan quality, *ii*) to compare two models which differ in the management of bilateral structure (as ipsi- and contra-lateral parotids), *iii*) to evaluate the stability of the model in varying the beam geometry and the dose fractionation, *iv*) to evaluate, with normal tissue complication probability (NTCP) estimations, if there should be an effective clinical benefit in planning with RP in place of the standard clinical planning procedures.

## Methods

### RapidPlan

The RapidPlan DVH estimation model configuration consists of two parts, as explained in literature [13]: 1) the plan and patient data modelling (data extraction from clinical database and subsequent model training); 2) the optimization objective choice (as DVH lines or points, and priorities).

### Patient selection for the RapidPlan model configuration

Eighty-three patients presenting advanced HNC, stage III-IV, treated from 2010 to 2014, were selected from the department database. Sixteen had nasopharyngeal, 41 oropharyngeal, 26 hypopharyngeal or laryngeal tumours. The patients were chosen as they had treatment plans clinically considered as optimal in terms of quality and critical structures sparing.

A CT-scan was acquired for each patient in supine position (immobilized with a thermoplastic mask), with 3 mm adjacent slice spacing. Clinical target volumes (CTV) for elective and boost regions were delineated according to internationally accepted guidelines [19]. An isotropic 5 mm margin was added to CTV to obtain the planning target volumes (PTV). PTVs were finally cropped 4 mm inside the body contour.

All the treatment plans were optimized for VMAT technique (in its RapidArc form), with two to four arcs and collimator angles according to the patient anatomy complexity, 6 MV beam quality. Patients were treated on one of the department linacs: Edge, TrueBeam, TrueBeamSTx, Clinac DHX, or Unique (Varian Medical Systems, Palo Alto, CA), equipped with Millennium 120-MLC (5 mm leaf width at isocenter) or HD-MLC (2.5 mm leaf width). Inverse planning used the Progressive Resolution Optimizer PRO, and final calculations were performed with the Anisotropic Analytical Algorithm AAA, as implemented in the Varian Eclipse treatment planning system (versions 8.9 to 11). Doses were prescribed for all patients in 33 fractions, to total doses of 69.96 Gy and 54.45 Gy to the boost and the elective PTV, respectively. Plan normalization was to the mean high dose PTV.

### The RapidPlan model configuration: the DVH estimation

The first part of the DVH estimation model configuration consists in the training phase of the plans selected for the matched structures (OARs). This process generates the mathematical parameters (through principal component analysis and regression models) relating the geometric and dosimetric features, which are then used for the DVH estimation.

The RP model was generated for the following OARs: spinal cord, brain stem, oral cavity, parotids, submandibular glands, larynx, constrictor muscles (inferior, middle and superior), thyroid, eyes.

Two models were generated with a different management of the parotids:

- Model HN_2Par: the two parotids in the plans were distinguished as ipsi- and contra-lateral parotids in the model. Their selection was based on the proximity to the high- or low-dose level target (for ipsi- and contra-lateral parotid, respectively);
- Model HN_1Par: one single parotid structure in the model included each one of the parotids in the plans. All the other model characteristics were identical to the HN_2Par model.

In order to constrain the healthy tissue not belonging to any of the delineated critical structures with the aim of lowering the dose bath, the body (patient volume in the planning CT dataset) was copied into a new structure, with all the targets subtracted (Boolean operation), and included in the model. The choice of this structure was driven by the easiness and universality of its generation.

The targets included in the configuration were: the boost PTV_boost, the elective volume PTV_elective defined as the whole PTV excluding the PTV_boost with a margin of 4 mm in the axial directions only (gantry rotation plane), and the PTV_all being the whole PTV receiving all the dose levels. This last structure should allow the algorithm to properly evaluate the OARs sub-volumes (that are related to the target volumes overlapping) for the training phase of the DVH estimation algorithm configuration.

### The trained model verification with model analytics

The verification of the DVH estimation was performed with the Model Analytics (MA) tool, developed by Varian and offered as a cloud service for analysing RP models. With this tool, any model trained in Eclipse can be inspected (no patient data are transferred) with respect to a bunch of statistical and dosimetric parameters. The MA output is structured in “suggestions” which might improve the model prediction power. These can be related to the presence of structures or patients candidate to be excluded from the model, to possible gaps in dose-volume features where to add data. The suggestions from MA were analysed case by case with a detailed inspection of the specific plans; the proposed potential outliers were evaluated in order to exclude or keep the structure in the model. In two cases, it was judged that the dose to a specific OAR could have been improved, and were re-planned; the new improved plans were included in the final model replacing the original ones.

In the following, the results will refer to the final model.

### The RapidPlan model configuration: the optimization objectives and priorities

The second part of the DVH estimation model configuration is the selection of the optimization objectives and their priorities. Line, upper and mean objectives and priorities were selected for each structure in the model according to Table 1.

**Table 1:** Objectives in the model

Target/Organ	Objective	Dose [Gy or % of the specific target prescription]	Volume [%]	Priority
PTV_boost	Upper	101%	0%	120
	Upper	100%	0%	120
	Lower	99%	100%	120
	Lower	100%	100%	110
PTV_elective	Upper	101%	0%	110
	Upper	100%	0%	110
	Lower	99%	100%	110
	Lower	100%	100%	110
PTV_all	Lower	100%	100%	0
Spinal Cord	Line	Generated	Generated	Generated
	Upper	Generated	0%	90
Brain Stem	Line	Generated	Generated	Generated
	Upper	Generated	0%	90
Oral Cavity	Line	Generated	Generated	Generated
	Mean	Generated		60
Parotids (HN_1Par)	Line	Generated	Generated	Generated
	Mean	Generated		70
Ipsilat Parotids (HN_2Par)	Line	Generated	Generated	Generated
	Mean	Generated		70
Contralat Parotids (HN_2Par)	Line	Generated	Generated	Generated
	Mean	Generated		70
Submandibulars	Line	Generated	Generated	Generated
Larynx	Line	Generated	Generated	Generated
	Mean	Generated		60
Constrictors	Line	Generated	Generated	Generated
Thyroid	Line	Generated	Generated	Generated
Mandible	Line	Generated	Generated	Generated
	Upper	Generated	5%	Generated
Eyes	Line	Generated	Generated	Generated
	Mean	Generated		Generated
Body-PTV	Line	Generated	Generated	Generated
	Upper	30% of boost dose	Generated	120
	Upper	50% of boost dose	Generated	120
	Upper	70% of boost dose	Generated	120

The structure PTV_all was added with a lower objective with no priority. The reason is to assign all the target structures as such in the optimization phase (the optimizer considers as target the structures where a lower objective is defined). In this way the Normal Tissue Objective (NTO) tool, which governs the dose fall-off outside the target volumes, will act only outside this PTV_all volume and will not attempt to reduce the dose in the 4 mm margin between the PTV_boost and PTV_elective.

For all the trained OARs, a line objective with generated priority was included.

For serial organs (spinal cord and brain stem), to enforce the sparing of the maximum dose, an upper objective was added with generated dose at a fixed volume of 0% (this point is located to the maximum dose of the estimated line objective), with a fixed priority of 90, that is most probably higher than the generated priority, and rather close to the priority assigned to the PTV_elective.

For parotids, oral cavity and larynx structures, an additional mean objective was added, with the dose value generated by the model, fixing the priority according to the table, with the intent of enhancing the sparing.

Regarding the uninvolved tissue (body structure deducted all the targets), other than the line objective with generated priority, three upper objectives corresponding to the 30%, 50% and 70% of the high dose prescription level were added, with generated volume, and priority set to 170, with the aim of lowering the medium dose levels. This is usually done in the current practice by adding dummy structures where the dose bath is likely to be maintained below the 50% dose level. The priorities for such structure were quite high relative to the other priorities, since those volumes are large, and one of the goals for this model was to use RP with no delineation of patient specific extra-structures.

The automatic NTO tool was also activated in the model to improve the dose conformity. The priority cannot be generated, and a fixed value of 280 was set (the higher the priority, the better the dose conformity).

The values of the fixed priorities included in the model were the result of iterative testing of the model performance on real patients (similarly to the refinement also proposed by Hussein et al. [15]). The values were tuned to obtain plans that at the best were compliant with our institutions’ acceptance criteria and strategies concerning the trade-offs between target coverage and specific critical structures sparing.

### The model validation and stability

To validate the model(s), a cohort of 20 similar patients was selected; 10 among those ones used for the model training and 10 additional cases not included in the model configuration (these cases were selected among a pool of clinical plans of very good quality). The actual clinical plans (CP) were compared with the RP generated plans. All plans were optimized for RapidArc technology on 6 MV beams.

The CPs were obtained with the usual clinical procedures which included the delineation of many ad-hoc structures for optimization (as for example shells around the target with defined thicknesses, dummy structures to lower the dose bath, or structures generated by the isodose levels to homogenise the dose in the target). Then the optimization objectives and priorities were defined, manually or using templates; their location in the dose-volume graph was manually adjusted, depending on the specific patient anatomy. Finally, the optimization process started, and was generally repeated several times to better tune the dose toward the final planning goals. CP were optimized with PRO and calculated with AAA (versions 8.9 to 11).

For RP generated plans, only two extra-structures were delineated: the first was the PTV_elective (as Boolean difference between the PTV_all and the PTV_boost with a 4 mm axial margin, as described above), the second was the copy of the body structure followed by the Boolean subtraction the PTV_all. The planning procedure included the DVH estimation and automatic objective generation according to the model. During the RP based optimisation, no changes of the objectives nor priorities were allowed in order to exclude any operator dependent bias. Contrarily, the Multiple Resolution levels during the optimization process were manually kept in hold status until the flattening of the cost function. This manual interaction was necessary due to the very fast progress of the multiple resolution levels, sometimes insufficient to guarantee the best and complete optimisation. To account for the possible discrepancies between the dose distribution computed during the optimization (with a simplified model) and with the full algorithm (AAA), a second optimization was run for the last two multiple resolution levels.

RP were optimized with the Photon Optimizer PO and calculated with AAA (version 13.6), for compatibility with the CP that were generated through AAA calculations.

CP and RP based plans were finally quantitatively compared.

In order to explore the model stability relative to different conditions, a number of RP were generated for each patient. The explored conditions were the arc geometry, the dose fractionation, and the parotid management in the model (separating or not the ipsi- and contra-lateral organs). The plans were as following:

- **RP**_**OR**_**2P**_**33**: RapidPlan, same arc geometry as in the CP, model HN_2Par, 33 fractions (69.96 and 54.45 Gy SIB)
- **RP**_**OR**_**1P**_**33**: RapidPlan, same arc geometry as in the CP, model HN_1Par, 33 fractions (69.96 and 54.45 Gy SIB)
- **RP**_**OR**_**2P**_**30**: RapidPlan, same arc geometry as in the CP, model HN_2Par, 30 fractions (66.0 and 54.0 Gy SIB)
- **RP**_**2A**_**2P**_**33**: RapidPlan, 2 arc geometry (collimator ±12–25°, X jaw setting equal to 15 cm), model HN_2Par, 33 fractions (69.96 and 54.45 Gy SIB)

### Data analysis

The following four comparative analysis were evaluated according to the above plans:

- *RapidPlan validation*: RP_OR_2P_33 vs. CP. This comparison aimed to evaluate the performances of the RP model relative to the CP.
- *RapidPlan stability with arc geometry*: RP_OR_2P_33 vs. RP_2A_2P_33. The performance of the RP model was evaluated for plans generated with a simpler geometry.
- *RapidPlan stability with fractionation*: RP_OR_2P_33 vs. RP_or_2P_30. This comparison aimed to evaluate to possibility to use a RP model generated with plans presenting the same dose fractionation, for a different dose fractionation.
- *RapidPlan stability with parotid separation in the model*: RP_OR_2P_33 vs. RP_OR_1P_33. This part compared the two models, the one trained with two distinct parotid structures (one ipsi- and one contra-lateral) and the one trained with only one parotid structure including both separate parotid glands.

To estimate if the dosimetric differences between the CP and the RP could improve the patient sequelae, the possible clinical benefit was evaluated through NTCP estimation. NTCP was calculated for some OARs using the NTCP Poisson-LQ model (relative seriality NTCP model [20]) implemented in the biological evaluation tool of the Eclipse treatment planning system, as well as a composite NTCP. The parameters used for NTCP evaluation were:

- Parotids: endpoint xerostomia, D50 = 46 Gy, γ = 1.8, α/β = 3 Gy, seriality = 1;
- Oral cavity: D50 = 39 Gy, γ = 3, α/β = 3 Gy, seriality = 0.5;
- Larynx and thyroid: endpoint necrosis, D50 = 78.8 Gy, γ = 4.8, α/β = 3 Gy, seriality = 0.66;
- Spinal Cord: endpoint myelitis, D50 = 68.6 Gy, γ = 1.9, α/β = 3 Gy, seriality = 4;
- Brain Stem: endpoint necrosis, D50 = 65.1 Gy, γ = 2.4, α/β = 3 Gy, seriality = 1.

## Results

### Evaluation of the RapidPlan model

**Fig. 1:**
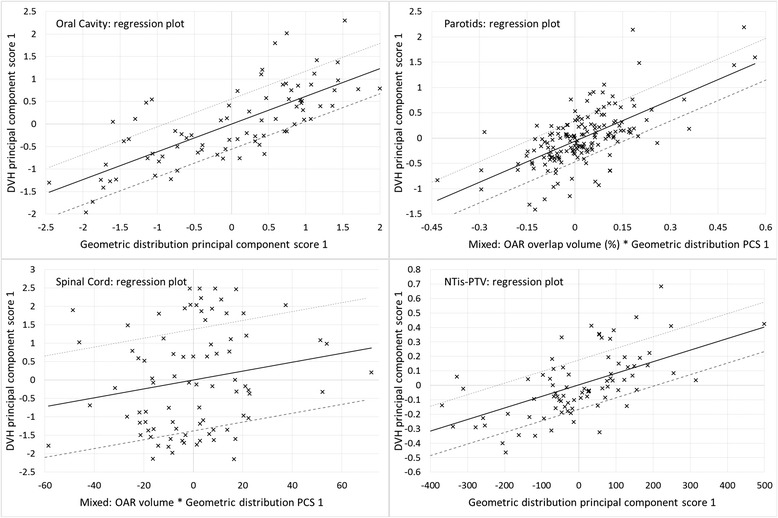
Regression plots

The model quality was evaluated checking the model goodness of fit statistics for each structure, with the coefficient of determination *R* ^2^ (between 0 and 1: the larger, the better) and the average Pearson’s chi square χ^2^ (the closer to 1, the better), and the model goodness of estimation with the mean squared error MSE between original and estimate (the closer to 0, the better). Those parameters, together with the number of trained structures and the number of potential outliers or influential points are reported in Table 2 for the most salient structures. Some regression plots are reported in Fig. 1.

**Table 2:** Evaluation of the model training

Structure	*R* ^2^	χ^2^	MSE	Trained structures	Potential outliers
Brain Stem	0.606	1.136	0.021	77	4
Constrictor inferior	0.659	1.120	0.028	32	9
Constrictor middle	0.507	1.133	0.028	25	4
Constrictor sup.	0.881	1.343	0.020	22	4
Eyes	0.743	1.111	0.007	36	8
Larynx	0.534	1.095	0.014	56	8
Mandible	0.747	1.056	0.005	71	3
NTis-PTV	0.568	1.066	0.0001	83	2
Oral Cavity	0.599	1.025	0.005	79	2
Parotid Contralat.	0.535	1.091	0.003	81	3
Parotid Ipsilateral	0.538	1.068	0.004	84	12
Parotids (sing.par model)	0.541	1.047	0.004	165	11
Spinal Cord	0.204	1.050	0.027	83	23
Submandibular	0.582	1.091	0.028	39	2
Thyroid	0.706	1.088	0.008	74	8

The potential outliers highlighted in the model log file, or pointed out in the MA tool were evaluated case by case. They were judged as not real outliers, being in the majority of the cases related to some anatomical differences with respect to the rest of the population in the model, all plausible and not anomalous anatomies (e.g. the parotid gland extending in some cases laterally to the masseter muscle).

The spinal cord structure presented the lowest coefficient of determination and the highest number of potential outliers (23, 3 for the Cook’s Distance larger than 10, indicating potential influential points, 20 for the modified Z-score larger than 3.5, indicating potential geometric outliers). No cases were considered negatively influential for the model. This structure was delineated case by case with no predefined strategy for its length, i.e. in some cases it was contoured only a couple of cm more caudal than the target, in some others more than 10 cm. This fact made the geometrical feature in the model to be a combination of the geometrical distribution principal component score 1 and the OAR volume; this last parameter, for a structure like the spinal cord that has not a defined volume (length), could in principle generate some unexpected DVH estimation results.

In general, after the careful visual check of the potential outliers/influential points and the comparison between the actual (model input) and estimated (model output) DVH, the model was considered acceptable for starting the validation process.

### RapidPlan validation

In Table 3 a summary of the dosimetric results as mean ± SD for some of the parameters are reported for the RP plans, as well as CP.

**Table 3:** Dosimetric results of all plans

Structure	Parameter	Clinical plan	RP_OR_2P_33	RP_OR_1P_33	RP_2A_2P_33	RP_OR_2P_30
PTV_boost	D_2%_ [%]	103.5 ± 0.9	103.2 ± 0.8	103.3 ± 0.8	103.6 ± 0.8	103.2 ± 0.8
			p_1_ = 0.058	p_2_ = 0.466	p_3_ = 0.003	p_4_ = 0.459
					p_5_ = 0.843	
	D_98%_ [%]	93.3 ± 1.6	94.4 ± 1.4	94.3 ± 1.5	93.6 ± 1.4	94.6 ± 1.5
			p_1_ = 0.002	p_2_ = 0.304	p_3_ < 0.001	p_4_ = 0.017
					p_5_ = 0.341	
PTV_elective	D_2%_ [%]	108.8 ± 5.2	105.7 ± 1.2	105.8 ± 1.2	106. ±1.1	105.1 ± 0.8
			p_1_ = 0.008	p_2_ = 0.272	p_3_ = 0.001	p_4_ < 0.001
					p_5_ = 0.038	
	D_98%_ [%]	93.6 ± 1.8	92.8 ± 1.8	92.8 ± 2.1	91.4 ± 1.5	92.3 ± 1.8
			p_1_ = 0.077	p_2_ = 0.986	p_3_ < 0.001	p_4_ < 0.001
					p_5_ < 0.001	
	Mean [Gy]	55.1 ± 0.6	54.4 ± 0.3	54.4 ± 0.4	54.4 ± 0.4	53.9 ± 0.3
			p_1_ < 0.001	p_2_ = 0.701	p_3_ = 0.366	
					p_5_ < 0.001	
Spinal Cord	D_max_ [Gy]	39.1 ± 7.3	32.0 ± 3.9	31.8 ± 3.9	33.1 ± 3.4	29.3 ± 3.2
			p_1_ < 0.001	p_2_ = 0.058	p_3_ = 0.001	p_4_ = 0.048
					p_5_ = 0.001	
Brain Stem	D_max_ [Gy]	37.9 ± 15.1	31.0 ± 13.4	31.1 ± 13.5	30.8 ± 13.5	29.8 ± 12.1
			p_1_ = 0.023	p_2_ = 0.757	p_3_ = 0.864	p_4_ = 0.515
					p_5_ = 0.022	
Oral Cavity	Mean [Gy]	45.4 ± 9.3	40.2 ± 11.1	40.2 ± 11.0	41.3 ± 10.5	38.2 ± 10.2
			p_1_ < 0.001	p_2_ = 0.839	p_3_ = <0.001	p_4_ = 0.013
					p_5_ < 0.001	
Parotids	Mean [Gy]	27.1 ± 6.0	24.9 ± 5.4	24.9 ± 5.2	25.2 ± 5.4	23.8 ± 4.9
			p_1_ < 0.001	p_2_ = 0.978	p_3_ = 0.063	p_4_ = 0.003
					p_5_ < 0.001	
Larynx	Mean [Gy]	35.6 ± 7.4	25.7 ± 5.5	25.8 ± 5.6	26.9 ± 5.8	24.8 ± 5.2
			p_1_ < 0.001	p_2_ = 0.734	p_3_ = 0.005	p_4_ < 0.001
					p_5_ < 0.001	
Thyroid	Mean [Gy]	45.6 ± 7.2	44.0 ± 6.6	44.1 ± 6.5	44.8 ± 6.4	42.0 ± 6.3
			p_1_ = 0.094	p_2_ = 0.542	p_3_ < 0.001	p_4_ < 0.001
					p_5_ = 0.358	
Eyes	Mean [Gy]	3.9 ± 3.3	2.9 ± 1.6	3.0 ± 1.6	3.1 ± 1.5	2.8 ± 1.4
			p_1_ = 0.036	p_2_ = 0.618	p_3_ = 0.253	p_4_ = 0.229
					p_5_ = 0.071	
Constrictors	Mean [Gy]	58.1 ± 12.3	55.2 ± 15.3	55.3 ± 15.2	55.7 ± 14.7	52.2 ± 14.6
			p_1_ < 0.001	p_2_ = 0.236	p_3_ = 0.006	p_4_ = 0.293
					p_5_ < 0.001	
Submandibulars	Mean [Gy]	63.9 ± 6.3	63.8 ± 6.8	63.8 ± 6.8	63.9 ± 6.8	61.0 ± 5.4
			p_1_ = 0.399	p_2_ = 0.598	p_3_ = 0.010	p_4_ < 0.001
					p_5_ = 0.866	
(Body-PTV)/PTV	V_20Gy/V_PTV	3.32 ± 0.48	2.74 ± 0.46	2.75 ± 0.47	2.67 ± 0.45	2.57 ± 0.44
			p_1_ < 0.001	p_2_ = 0.125	p_3_ = 0.009	
					p_5_ < 0.001	
	V_35Gy/V_PTV	1.46 ± 0.28	1.07 ± 0.24	1.08 ± 0.24	1.07 ± 0.22	0.97 ± 0.21
			p_1_ < 0.001	p_2_ = 0.002	p_3_ = 0.881	
					p_5_ < 0.001	

p_1_ = *p*-value between RP_OR_2P_33 and CP (RapidPlan)

p_2_ = *p*-value between RP_OR_1P_33 and RP_OR_2P_33 (different model)

p_3_ = *p*-value between RP_2A_2P_33 and RP_OR_2P_33 (arc geometry)

p_4_ = *p*-value between RP_OR_2P_30 and RP_OR_2P_33 (fractionation)

p_5_ = *p*-value between RP_2A_2P_33 and CP (arc geometry)

**Fig. 2:**
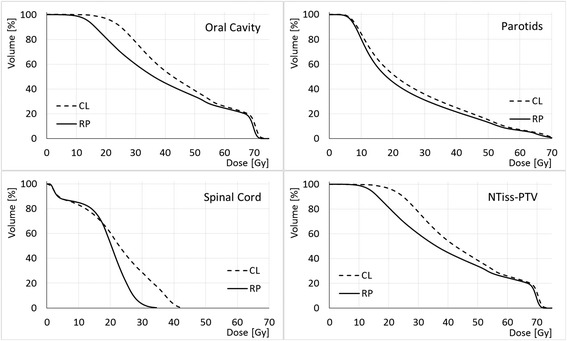
Mean DVH for some OAR, for CP and RP_OR_1P_33

**Fig. 3:**
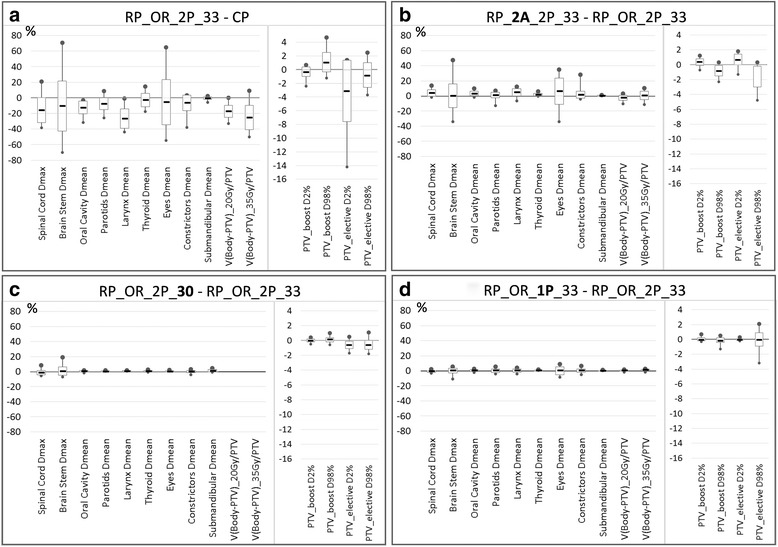
Percentage differences of dosimetric parameters. **a** RP vs. CP in the same conditions; **b** beam arrangement stability; **c** dose fractionation stability (in this plot the percentage differences refer to the relative doses); **d** bilateral structures: models trained with single or separate structures

The RP validation consisted in the comparison between RP_OR_2P_33 plans and the CP. Figure 2 presents a comparison of some OAR DVHs, and Fig. 3 shows the percentage differences of dosimetric parameters, between RP and CP relative to CP values for the OARs and targets. RP in average improved significantly the CP for all the OARs, with mean differences of 27% (10 Gy) for the larynx mean dose, 12% (5 Gy) for the oral cavity mean dose, 8% (2 Gy) for the parotids mean dose. p values are also reported in Table 3. Regarding the targets, RP increased the target homogeneity relative to CP. The goal to PTV_elective of a mean dose of 54.45 Gy (prescription) was achieved by the RP, with an average of 54.4 ± 0.3 Gy, while the CP average value was 55.1 ± 0.6 Gy, 1.3% higher than expected.

### RapidPlan stability with arc geometry

The dosimetric results are summarized in Table 3 and Fig. 3b. Differences between RP generated using the original clinical arc setting and with a simpler technique of two arcs were in general statistically significant, with a plan quality deterioration when a simpler technique was used. However, the variations were, in absolute values, quite small: 5 and 3% (~1Gy) for the larynx and oral cavity mean doses, respectively, 1% (<0.5 Gy) for the parotid mean dose. In addition, the target homogeneity presented a dose spread of 1.2 and 2.2% larger for the boost and elective volume, respectively, when the simpler arc arrangement was used. However, these differences are to be ascribed to the lower degrees of freedom of the two arcs relative to a more complex geometry, which allowed a better dose optimization. Conversely, the RP model showed to be stable in the DVH estimation and consequent optimization objective generation, leading to similar results for different beam geometries.

### RapidPlan stability with fractionation

To compare the differences in the plan quality for plans generated for different dose prescriptions, the analysis was based on the relative (percentage) difference between the two fractionations (30 or 33 fractions to deliver 66.0 or 69.96 Gy to the boost volume). This was also consistent with the model training, that uses the plan dataset in terms of dose relative to the plan prescription, which is set as 100% level.

The differences of few percent (Table 3) were consistent with expectations; Fig. 3c presented the data as percentage differences of the relative (to the prescription) doses. Some more variation was for the two serial organs, where the maximum dose was recorded, while the line objective acts more on the entire DVH (that is the reason why in the model the maximum dose objectives for those structures have been added with rather high priority).

### RapidPlan stability with parotid separation in the model

As shown in Table 3 and Fig. 3d, the two models trained with one single parotid structure or two separated ipsi- and contra-lateral parotid did not present significant differences, neither in the parotid sparing, nor in the other structures, OAR sparing or target coverage. In this case, the differences are in average less than 0.5%, not significant. This result suggests that it might be not necessary to distinguish between ipsi- and contra-lateral structures. This result was not evident from the regression model, since the ipsi- and contra-lateral parotids had different geometric feature for DVH estimation in the model (the combination of the OAR volume overlapping the target and the principal component score 1 for the ipsi-lateral, and only the principal component score 1 for the contra-lateral).

### NTCP evaluation

Conscious that the estimated probability values cannot be considered as such, the NTCP data was analysed in a relative way. The spinal cord and brain stem plans did not reach the tolerance level, and the very low NTCP values, if not zero, did not give any significant difference between CP and RP (RP_OR_2P_33). Not significant were also the NTCP differences for larynx and thyroid.

Different are the cases of the parotids and the oral cavity, where reductions of about 2 and 8% in NTCP with the RP solution presented p values of 0.002, and 0.001, respectively. The composite NTCP estimation was about 7% higher for CP, with *p* = 0.001.

Those values indicated that a toxicity profile difference could be observed in clinical practice when RP planning is going to be used in place of CP.

## Discussion

A KBP RP model with 83 patients has been generated for advanced HNC VMAT planning. With knowledge-based optimisation, individualised dose-volume objectives can be derived automatically and reliably from earlier good clinical experience via mathematical models. The avoidance of pre-defined and fixed constraints would allow to possibly improving the dose distributions for each patient to their maximum and/or to showing irreducible conflicts requiring the acceptance of higher doses to the OAR or the change of the treatment strategy. The choice of the type and value of the optimization objectives is of great importance. The key is to maximise the use of parameters generated by the model rather than enforcing fixed dose-volume constraints which would reduce the KBP process to nothing more than an advanced template. To exemplify, it is possible that for a given patient the KBP model could predict, e.g., a mean parotid dose much lower than the clinical aim of 25 Gy. An optimisation driven by templates might fail in achieving the maximal parotid sparing while the KBP system, properly used, would be capable to maximise the sparing.

Concerning the main aims of the study, the RP KBP approach was shown to be robust with respect to: i) the use (or not use) of separated ipsi- and contra-lateral parotids; ii) the beam geometry; iii) the fractionation. Those specific points were not yet intensively analysed in the literature.

Regarding the use of ipsi- and contra-lateral structures, the results indicated that this is not significant, and the dose differences noticed were of very small values. This was indeed expected, since the possible higher doses to the parotids were due to the organ volume overlapping the target volume, and this feature is included in the model. The 1-parotid model would allow a simplification of the model (all the parotids would be matched to the same structure), increase the power of the organ specific training since the number of structures in the model would be doubled, and finally ease the use of the model in the routine practice, where there is no need to choose for each parotid which is the correct structure to match with.

Concerning the fractionation, the scheme of 66 Gy in 30 fractions was adopted for the RP_OR_2P_30 plans whose optimisation was based on a model trained with plans generated to deliver 69.96 Gy in 33 fractions. The results were fractionation independent, i.e. the plans obtained for higher or lower total doses resulted with organs at risk sparing proportional to the total delivered dose, as expected. From this point, a simple consideration could lead to the concept that it could be better to generate a model with high quality plans delivering high prescription doses, since in the case of altered fractionation with lower doses (hypofractionation) the critical structures would receive lower physical doses. It is important to consider that the biological effective dose is not taken into account for DVH estimation.

Concerning the RP robustness relative to the beam geometry, the model was trained using cases with an average of 3.5 ± 0.8 arcs (ranging from 2 to 4) per plan, while it was validated also using a simpler 2-arc geometry, equal for all patients. The significant difference between the RP with the clinical and the 2-arc geometry was generated by the obvious fact that more arcs can give better plan quality (p_3_ values in Table 3). However, in the same table the p_5_ values resulting from the comparison between the CP and the RP with 2-arc geometry confirmed the significant superiority of the RP relative to the CP for OAR sparing, although using a simplified and penalising geometry. Similarly to the application of RP with different beam geometries, the possibility to use different techniques (IMRT and/or VMAT) or different patient positioning (supine or prone) have been recently explored by two groups. Hussein et al. [15] reported about IMRT as well as VMAT plans from a RP model based on IMRT only plans, resulting in improved plan quality for both techniques. Wu et al. [21] validated the usability of a RP model based on VMAT plans on rectal cancer patients positioned in supine setup, in a set of patients in supine or prone position, optimizing IMRT plans. They reported a consistent higher plan quality for RP plans for both patient orientations, while they found the need to readapt the target objectives when IMRT plans were optimized, relative to VMAT plans.

Primarily two groups performed the validation of RP use in the treatment planning of HNC patients. Tol et al. [11] evaluated models trained with 60 plans or with subgroups of 30 plans of patients planned with VMAT technique, while Chang et al. [12] reported about a model trained with 79 plans of patients treated for nasopharyngeal cancer with IMRT technique. In both cases the authors reported no indications concerning the objective selection in terms of values (generated by the model and the knowledge, or fixed) and priorities (generated or fixed), making difficult to fully compare those models with what presented in the current work. However, the choice of the objectives, and the proper use of the generated or fixed constraints and priorities would change the results of the model. The objectives here presented in Table 1 were the result of an intensive testing phase of model refining.

Two publications [15, 18] reported about the dosimetric impact of the outliers or the influential points in the model configuration. The authors concluded that outlier removal had only marginal or no effect on the resulting plan quality. This is what we experienced also in the current work, where no potential outliers, after careful check of the causes that could have brought to mark them as such, were excluded from the model training.

An improvement was found in the current study when an explicit mean-generated objective dose was added other than the line dose objective. This could be caused by the fact that, in the current RP implementation (version 13.6), the mean dose generated by the DVH estimation engine refers to an OAR sub-volume, while, during the optimization phase, this objective is applied to the entire structure. On one side this effect would improve the structure dose sparing, on the other side the balance between the trade-offs is going to be modified and distorted toward a higher OAR sparing at a price of a possible lower target coverage, in part depending on the specific patient anatomy and not only to the intended planning strategies.

The dose calculation algorithm is a critical point in any plan quality comparison. In particular, for advanced HNC treatments, often some air cavities are included in the target delineation. This would fictitiously lower the target coverage when advanced dose calculation algorithms, like Acuros, are used. In fact, the dose deposited inside air cavities is very low, and this is better estimated by Acuros relative to AAA [22, 23], although showing a lower target coverage. In the current work, we decided to keep, in the validation phase, the same dose calculation algorithm that was used for the CP, in order to make the plan comparison fair, excluding the differences related to the different algorithms. However, the dose calculation algorithm issue needs to be highlighted for the plans selected for a RP model. Of course, a better dose estimation should be preferred. This would entail the need, for consistency reasons, to precisely define the target delineation rules; it is clear that air cavities, where there is no mass to irradiate, should not be part of the target delineation. The awareness of a possible lower delineated-target coverage is left to the planning phase, which is not part of the automation planning process, with small adjustments tailored to each specific patient. What is important in the RP model configuration and its subsequent clinical use is indeed the consciousness of the instrument, to better drive the specific optimisation.

A possible limitation of the RP management of the targets objectives in the model is the maximum number of allowed structures labelled as target in the model, currently limited to three. In principle, the use of the PTV_all as we managed in this study, would limit to two the number of dose levels of the SIB. This is our current practice, but it is also common to plan SIB with three dose levels (as e.g. according to UCSF trial [24]). In such a case, the limitation can be however overcome by assigning, for example, the low and intermediate risk targets to the same structure in the model. During planning, both the low and intermediate risk target structures would be matched to the single structure in the model with a certain prescription dose; then, manually, the dose levels specific of each of the target structures would be adjusted. In this way, the limitation of the model to host only three targets does not forbid the RP use with a higher number of targets.

The plan quality in the clinical practice depends on the planner’s skills. By consequence, the patient selection for the RP model preparation is influenced by this fact, and this is a general limitation of the present study and of any RP model generation. With the aim of minimizing the planner influence, the 83 plans selected for the model train, as well as the additional 10 patients for the validation process, were chosen with no attention to the planner, but only to the plan quality (in our pool of patients the plans were distributed among all the clinical planning team). However, though very small, the human factor might play a role in the study results.

From the here presented results, as well as from all the publications showing an improvement in the RP plan quality, a new model trained with the KBP optimized plans, could produce plans of even better quality, up to the physical limitations of delivery and trade-offs. This next step is still under investigation within our group.

## Conclusions

The RP HNC model here presented showed improved plan quality relative to the clinical plans, stable results for beam geometry, management of bilateral critical structures, and dose fractionation. An adequate choice of the objectives in the model is necessary for the trade-offs strategies. A possible clinical benefit as reduced toxicity remains to be tested.

## Abbreviations

AAA: Anisotropic analytical algorithm
CP: Clinical plan
CTV: Clinical target volume
DVH: Dose-volume histogram
HNC: Head and neck cancer
IMRT: Intensity modulated radiation therapy
KBP: Knowledge-based planning
MA: Model analytics
MSE: Mean squared error
NTCP: Normal tissue complication probability
NTO: Normal tissue objective
OAR: Organ at risk
PO: Photon optimizer
PRO: Progressive resolution optimizer
PTV: Planning target volume
RP: RapidPlan
SIB: Simultaneous integrated boost
VMAT: Volumetric modulated arc therapy
